# Multiple Endocrine Neoplasia Type 1: The Potential Role of microRNAs in the Management of the Syndrome

**DOI:** 10.3390/ijms21207592

**Published:** 2020-10-14

**Authors:** Simone Donati, Simone Ciuffi, Francesca Marini, Gaia Palmini, Francesca Miglietta, Cinzia Aurilia, Maria Luisa Brandi

**Affiliations:** 1 Department of Experimental and Clinical Biomedical Sciences “Mario Serio”, University of Study of Florence, Viale Pieraccini 6, 50139 Florence, Italy; simone.donati11@gmail.com (S.D.); simone.ciuffi@unifi.it (S.C.); francesca.marini@unifi.it (F.M.); gaia.palmini@unifi.it (G.P.); francesca.miglietta91@gmail.com (F.M.); aurilia.cinzia@gmail.com (C.A.); 2 Unit of Bone and Mineral Diseases, University Hospital of Florence, Largo Palagi 1, 50139 Florence, Italy; 3 Fondazione Italiana Ricerca Sulle Malattie Dell’Osso (FIRMO Onlus), 50141 Florence, Italy

**Keywords:** MEN1, parathyroid glands, pituitary gland, GEP-NETs, miRNAs, circulating miRNAs, non-invasive biomarkers, personalized medicine

## Abstract

Multiple endocrine neoplasia type 1 (MEN1) is a rare inherited tumor syndrome, characterized by the development of multiple neuroendocrine tumors (NETs) in a single patient. Major manifestations include primary hyperparathyroidism, gastro-entero-pancreatic neuroendocrine tumors, and pituitary adenomas. In addition to these main NETs, various combinations of more than 20 endocrine and non-endocrine tumors have been described in MEN1 patients. Despite advances in diagnostic techniques and treatment options, which are generally similar to those of sporadic tumors, patients with MEN1 have a poor life expectancy, and the need for targeted therapies is strongly felt. MEN1 is caused by germline heterozygous inactivating mutations of the *MEN1* gene, which encodes menin, a tumor suppressor protein. The lack of a direct genotype–phenotype correlation does not permit the determination of the exact clinical course of the syndrome. One of the possible causes of this lack of association could be ascribed to epigenetic factors, including microRNAs (miRNAs), single-stranded non-coding small RNAs that negatively regulate post-transcriptional gene expression. Some miRNAs, and their deregulation, have been associated with MEN1 tumorigenesis. Recently, an extracellular class of miRNAs has also been identified (c-miRNAs); variations in their levels showed association with various human diseases, including tumors. The aim of this review is to provide a general overview on the involvement of miRNAs in MEN1 tumor development, to be used as possible targets for novel molecular therapies. The potential role of c-miRNAs as future non-invasive diagnostic and prognostic biomarkers of MEN1 will be discussed as well.

## 1. Introduction

Multiple endocrine neoplasia type 1 (MEN1) (OMIM #131100) is a rare inherited tumor syndrome, characterized by the occurrence of multiple neuroendocrine tumors (NETs) in a single patient. Its major manifestations include primary hyperparathyroidism, gastro-entero-pancreatic neuroendocrine tumors (GEP-NETs), and pituitary adenomas, originally referred to as P-triad by Werner [1]. In addition to these, other MEN1-associated endocrine (i.e., adrenocortical tumors, and carcinoids) and non-endocrine (i.e., facial angiofibromas, collagenomas, lipomas, meningiomas) tumors have been described in MEN1 patients [2,3].

The estimated worldwide prevalence of MEN1 is approximately one in 30,000 individuals; syndrome distribution is equal between genders and no ethnic group or racial predilection has been reported [4]. MEN1 syndrome can occur either as sporadic form (10% of cases), when a family history of the disease is missing and only one affected individual is primarily identified, or familial form which occurs in 90% of cases [5]. A diagnosis of familial MEN1 is defined for an individual who has at least one first-degree relative with one or more of main MEN1-associated tumors and a *MEN1* disease-causing germline mutation [5].

MEN1 is a Mendelian syndrome, caused by inactivating mutations of the *MEN1* gene at 11q13, encoding a ubiquitously expressed 610-amino acid protein, named menin [6]. The inheritance is autosomal dominant and offspring have a 50% risk of inheriting the causative mutation, independent of gender [7]; the clinical penetrance is complete by the age of 50. Germinal heterozygous loss-of-function mutations of the *MEN1* gene lead to the development of MEN1 tumors by following Knudson’s “two-hit” hypothesis for classical tumor suppressor genes [5,8].

DNA testing and genetic counseling play a fundamental role in the management of MEN1 patients, since genetic screening allows the early identification of *MEN1* mutation carriers, in mutated pedigrees, in a presymptomatic stage, and enables the possibility to plan a life-long diagnostic program for the early identification of MEN1-associated tumors.

Treatment of MEN1-associated tumors is prevalently by surgery and generally similar to their counterpart tumors occurring in non-MEN1 patients, even though the treatment outcomes are not as successful as those in the sporadic forms, because of their multiple and more aggressive nature, the concurrence of metastases, and the genetic-driven post-surgical recurrences [6]. Despite recent advances in the diagnosis and treatment of MEN1 syndrome, patients with MEN1 have a poor life expectancy. The primary purpose is to maintain a good patient quality of life by keeping the individual disease- and symptom-free for as long as possible and intervening at early stages of tumors to prevent possible malignant progression and metastases development. Particularly noteworthy is the absence of correlation between *MEN1* mutations and the clinical presentation of the syndrome, which does not permit the individual manifestation and progression of MEN1-associated tumors to be foreseen [1]. Consequently, genetic analysis cannot drive the design of a personalized clinical management of *MEN1* mutation carriers.

One of the possible causes of the lack of a direct genotype–phenotype correlation could be ascribed to epigenetic factors, acting as cofactors to the genetic mutation in driving the individual MEN1 tumorigenesis. Epigenetics is a reversible, phenotypic inheritance mechanism of transcription regulation, which is independent of DNA nucleotide sequence changes, and includes tissue-specific regulatory processes of gene expression, such as DNA methylation, histone modifications, and gene silencing by noncoding RNAs (ncRNAs) [9]. Alteration of one or more epigenetic mechanisms can be responsible for disease occurrence, including tumors, being suitable molecular targets for therapies and/or diagnostic and prognostic biomarkers in human diseases [10].

Among epigenetic factors, microRNAs (miRNAs) have been shown to be frequently deregulated in tumors, including breast, liver, lung, colorectal, ovarian and prostate cancers, leukemia, and other malignancies [11]. Studies have also shown that a specific miRNA signature could aid in discriminating normal tissue from pathological conditions [12], allowing these molecules to gain increasing attention as possible diagnostic and prognostic biomarkers in human tumors.

miRNAs are an evolutionary conserved class of small non-coding RNAs (approximately 18–25 nucleotides) that negatively regulate post-transcriptional gene expression [13]. It is estimated that at least 20–30% of human transcripts are regulated by miRNAs [13], principally by their direct interaction with the 3′ untranslated region (3′-UTR) of mRNA target, which induces either mRNA degradation or protein translational inhibition, via complete or incomplete complementarity, respectively [14,15,16]. It has also been reported that they may bind with other regions, including 5-UTR, coding sequence, and gene promoters [14]. Currently, there are 2693 Homo sapiens mature miRNAs registered in the miRBase registry [miRBase. Available online: http://www.mirbase.org (accessed on 20 Sept 2020); release 22.1: October 2018] [17].

miRNAs are indicated by a sequential number, accordingly to their progressive discovery. The first three letters before the name of the miRNA indicate the organism where it was found (i.e., “hsa”, “mmu”, and “rno” for human, mouse, and rat, respectively). Lettered suffixes differentiate closely related mature sequences, derived from distinct genetic loci (i.e., miR-121a and miR-121b). Previously, a mature sequence known to be predominantly expressed was referred to as a mature or guide strand, while the less expressed miRNA (passenger or star strand) was marked by an asterisk (i.e., miR-56*). In the last releases, miRBase replaced it with the suffixes -3p and -5p that indicate the two different mature forms of a miRNA, expressed, respectively, from the 3’ and the 5’ arms of the precursor miRNA (i.e., miR-142-3p and miR-142-5p) [18].

Altered expression of specific miRNAs has been reported between normal, benign, and malignant tissues of MEN1-associated NETs, including pituitary [19,20,21], parathyroid [22,23], and GEP tract [24,25] tumors. Results from a previous study of our Research Group showed that menin is a direct target of miR-24-1, and that this miRNA may contribute to MEN1 parathyroid tumorigenesis [26]. In particular, after the first inherited germinal “hit”, the somatic onset and progression of tumors could be under control of an epigenetic “negative feedback loop” between miR-24-1 and menin, which mimics the second hit of Knudson’s model of tumorigenesis. In a subsequent study, we identified three additional miRNAs (i.e., miR-4258, miR-1301, and miR-664) as involved in MEN1 parathyroid neoplasia, by directly targeting tumor suppressor genes, known to be associated with the development of different familial forms of parathyroid tumors [27].

In addition to their common cellular localization, cell-free miRNAs were detected in a plethora of extracellular biofluids, including cerebrospinal fluid [28], saliva [29], breast milk [30], urine, tears, bronchial lavage, seminal fluid [31], and ovarian follicular fluid [32]. Conversely to cellular RNA species, the cell-free miRNA molecules are remarkably stable in biological fluids, despite a high RNase activity, and able to withstand even in deleterious conditions (e.g., boiling, multiple freeze–thaw cycles, and high or low pH) [33,34]. Their increased stability in biological fluids is generally thought to be attributable to binding with specific proteins, especially Argonaute 2 (Ago2), high-density lipoproteins (HDLs), and nucleophosmin 1 (NPM1) [35,36,37], or encapsulation in membrane-bound vesicles, such as microvesicles (1 μm), exosomes (50–90 nm), and apoptotic bodies [29,38].

In 2008, four independent studies demonstrated not only the existence of stable cell-free mature miRNAs in blood, but also that changes in these circulating miRNAs (c-miRNAs) expression profiles can be indicative of both physiological and pathological states, thus making these candidates non-invasive biomarkers for a wide range of diseases [33,34,39,40]. An altered c-miRNA expression profile has been demonstrated for different disease subgroups, such as cancer, viral infections, cardiovascular and muscular disorders, nervous system disorders, and diabetes [41].

The involvement of c-miRNAs as a novel way of intercellular communication is now established. In accordance with a proposed model, these molecules would be secreted and transferred to recipient cells, where they act by regulating host cell activity. In particular, it has been proposed that vesicle-bound miRNAs may enter cells by endocytosis, phagocytosis, or direct fusion with cell plasma membranes, while vesicle-free miRNAs may be taken up by specific receptors on the host cell surface [35,42,43]. However, the mechanisms by which miRNAs are secreted and taken up by cells, and their biological function, are not well understood and require further investigation.

The identification of specific expression profiles of miRNAs specifically characterizing neuroendocrine tumor cells in MEN1 patients is an important step for the future development of target RNA antagomirs-based strategies to control the MEN1 tumorigenesis. In addition, the recognition of c-miRNAs, specifically associated with different clinical MEN1 phenotypes, would be useful for the identification of novel diagnostic molecules, to be used together in the pre-existing biochemical and instrumental approaches.

The aim of this review is to provide a general overview on the involvement of intracellular miRNAs in the development and progression of MEN1-associated tumors, which could be used as possible targets for novel molecular therapies.

First, we conducted a systematic literature search to select all studies regarding miRNAs involved in human endocrinopathies of the three main neuroendocrine tissues affected in MEN1 syndrome. The research was focus on papers published over the last five years (2015–2020), and performed by using different combinations of relevant keywords: “miRNAs”, “pituitary gland”, “GEP-NETs”, “parathyroid glands”, “NETs”. In addition, a specific overview of literature on “miRNA and MEN1” was performed, including both human and animal studies.

The potential role of c-miRNAs as future non-invasive diagnostic and prognostic biomarkers of MEN1 will also be discussed.

## 2. miRNAs in Parathyroid Tumors

After reports of the importance of the miR-371-373 cluster in embryogenesis, development, and tumorigenesis of parathyroid tumors, Verdelli et al. [44] explored the role of one of its members, miR-372, in this pathology. They found that the expression levels of miR-372 were significantly increased in most parathyroid adenomas (PTAs), atypical PTAs, and parathyroid carcinomas (PTCs) compared to normal glands. In situ hybridization analysis carried out on PTA cells showed that miR-372-positive parathyroid tumor cells were scattered throughout the tumor parenchyma. In PTA-derived cells, transfected with miR-372 mimic, authors observed that this miRNA reduced the expression of its targets, CDKN1A/p21 and LATS2, both at mRNA and protein levels. Although the viability of parathyroid cells was not affected by miR-372 overexpression, this miRNA protected primary PTA-derived cultures from chemotherapeutic agent-induced apoptosis. Parathormone (PTH) mRNA levels were positively modulated by miR-372 overexpression in parathyroid tumor cells, while the Wnt pathway was inhibited in the miR-372-expressing parathyroid tumor cells, through the miR-372-induced upregulation of DKK1.

Establish a diagnosis of PTC is difficult in the absence of obvious metastasis, local invasion, or recurrence. To verify if an miRNA signature able to distinguish between PTC and PTA exists, Hu et al. [45] examined miRNA expression levels between tissue samples of 17 PTCs and 41 sporadic PTAs. Expression levels of miR-222 were significantly upregulated, while those of miR-139, miR-30b, miR-517c, and miR-126* were significantly downregulated in PTCs with respect to PTAs. Receiver-operating characteristic (ROC) analysis indicated that the combination of miR-139 and miR-30b was the most powerful in distinguishing between PTCs and PTAs. Spearman correlation showed that serum calcium and intact PTH (iPTH) levels were respectively correlated with expression levels of miR-139 and miR-30b, miR-139, miR-222, miR-517c, and miR-126*. In addition, the expression levels of miR-30b was significantly negatively correlated with serum calcium, iPTH, and alkaline phosphatase (ALP) levels. These data suggest that a combination of miR-139 and miR-30b could be a promising diagnostic biomarker for PTCs. Validations of these preliminary results are requested in additional independent patient cohorts before reaching definitive conclusions.

Parathyroid tumors can occur as sporadic cancer or as hereditary syndromic or non-syndromic diseases. Hwang et al. [46] evaluated whether miRNA profiling could be a biomarker for distinguishing sporadic from hereditary parathyroid tumors. They analyzed miRNA expression in three sporadic PTAs, three MEN1-related PTAs, and two normal parathyroid tissue (NPT) samples. The results showed that nine miRNAs were differentially expressed between sporadic and hereditary parathyroid tumors vs. NPTs. After a Real-Time Quantitative Reverse Transcription PCR (qPCR)-based validation on 25 sporadic and 12 hereditary parathyroid tumors and 24 NPTs, only the miR-199b-5p was confirmed to be differentially expressed between the two different parathyroid tumor types. When compared to NPT tissue, miR-199b-5p expression was significantly downregulated in sporadic adenomas and upregulated in the hereditary form. Area Under the Curve (AUC) value, obtained by ROC analysis, confirmed the diagnostic potential of miR-199b-5p in distinguishing between sporadic and hereditary parathyroid tumors. In addition, there was a negative correlation between miR-199b-5p and PTH levels in sporadic PTAs, whereas no significant correlation was observed in the hereditary counterparts. In silico target prediction software revealed that targets of this miRNA were significantly associated with pathways in tumorigenesis, focal adhesion, and central carbon metabolism in cancer. Therefore, miR-199b-5p appears to be a suitable diagnostic biomarker for distinguishing these two forms of PTAs.

Only few studies have been specifically conducted on miRNA involvement in MEN1 PTAs.

In 2012, Luzi et al. [26] were the first to demonstrate a direct link between miR-24-1 and menin, with miR-24-1 acting like an oncomir, blocking menin expression and being, thus, responsible for MEN1 parathyroid tumorigenesis via an epigenetic mechanism mimicking the “Knudson’s second hit” at somatic level. Expression levels of miR-24-1, MEN1 mRNA, and menin protein were all studied in eight PTA tissues from *MEN1* gene mutation carriers (four presenting somatic loss of heterozygosity (LOH) of *MEN1* locus and missing the second wild type copy of the gene and four without MEN1 locus LOH and, thus, maintaining one wild type MEN1 allele), in three sporadic non-MEN1 PTAs, and in one NPT. PTAs from MEN1 patients with loss of both MEN1 alleles showed no expression of MEN1 mRNA, menin, and miR-24-1. PTAs from MEN1 patients maintaining one wild type copy of the *MEN1* gene showed only a reduced expression of MEN1 mRNA, absent expression of menin, and an overexpression of miR-24-1, suggesting that the increased expression of this miRNA is directly responsible for the post-transcriptional block of menin translation, acting in silencing the expression of wild type menin, as an epigenetic oncogenic factor, and inducing parathyroid tumorigenesis before the MEN1 LOH occurrence.

Later, the same research group [47] confirmed, by in vitro functional studies, the existence of an autoregulatory negative feedback-loop between miR-24-1, MEN1 mRNA, and menin, exerting a key role in MEN1 tumorigenesis.

More recently, Luzi et al. [27] further investigated the possible involvement of miRNAs in MEN1 parathyroid tumorigenesis by profiling the expression of 1890 human miRNAs in seven PTAs (four with somatic LOH at the *MEN1* locus and three still retaining, at somatic level, one wild type copy of the *MEN1* gene) with respect to two sporadic PTAs from non-MEN1 individuals, used as controls. According to the fold change (FC) >1.5 and *p*-value < 0.05, authors identified eight differentially expressed miRNAs in non-LOH MEN1 PTAs and two differentially expressed miRNAs in LOH MEN1 PTAs, compared to the control group. In addition, six miRNAs resulted to be significantly differentially expressed in LOH MEN1 PTAs vs. non-LOH MEN1 PTAs. Following qPCR validation, authors observed that the expression levels miR-4258, miR-664, and miR-1301 were significantly differentially expressed in these two groups. miR-4258 was remarkably downregulated in LOH MEN1 PTAs compared with non-LOH MEN1 PTAs, while its expression was upregulated in non-LOH MEN1 PTAs compared with the control group. miR-664 was upregulated in non-LOH MEN1 PTAs and down-regulated in LOH MEN1 PTAs, both vs. control group. Finally, miR-1301 resulted to be upregulated in LOH MEN1 PTAs with respect to controls. In silico analysis showed that these three miRNAs directly targeted genes associated with the development of different inheritable forms of parathyroid tumors (i.e., *CCND1*, *RET*, *CDKN1B*, *RB1*, *VDR*, *PRDM2*, *CDKN2C*, and *CDC73/HRPT2* genes). All these data indicated these three miRNAs as potentially involved in MEN1 parathyroid tumorigenesis, revealing them as potential tumor biomarkers to be used as molecular targets for therapies.

Grolmusz et al. [48] also investigated MEN1-targeting miRNAs as a possible underlying cause of menin deficiency in MEN1-associated and sporadic primary hyperparathyroidism (PHPT). Germinal *MEN1* gene mutations were identified in all 14 patients; somatic *MEN1* gene mutations were present in 25% of sporadic PHPTs tissues. Nuclear menin expression was absent in all parathyroid specimens from MEN1 PHPT patients and in 11 out of 40 sporadic PHPT-derived parathyroid tissues. Among the 35 miRNAs predicted to bind to the 3′ UTR of *MEN1* gene through in silico analysis, six miRNAs (miR-24, miR-28, miR-326, miR-484, miR-637, and miR-744) were concordantly predicted by at least two target prediction algorithms and were selected for subsequent analysis in qPCR. The expression levels of miR-24 and miR-28 were upregulated in sporadic compared with MEN1-associated PHPT tissues, while no differences were observed in miRNA expression levels between menin-positive and menin-negative PHPT tissues. This study confirmed that germline and somatic *MEN1* mutations resulted in a total lack of menin expression in most PHPT tissues and indicated that miR-24 and miR-28 could have a critical role in the pathogenesis of PTA-derived PHPT.

Table 1 summarizes information about differentially expressed miRNA in parathyroid glands.

## 3. miRNAs and GEP-NETs

GEP-NETs are a heterogeneous group of neoplasms that arise from different neuroendocrine cells diffuse in various organs of the gastro-entero-pancreatic tract.

Two studies evaluated whether specific miRNA expression profiles correlated with different anatomic sites of origin of GEP-NETs, and can be, thus, used for classifying and grading well-differentiated GEP-NETs.

In the first study [49], expression screening of different GEP-NET specimens revealed that in NETs of primary pancreas, ileum, appendix, and rectum, there were, respectively, 13, 9, 4, and 1 differentially expressed miRNAs. In addition, seven miRNAs resulted differentially expressed in metastatic tumors (both for nodal and distant metastases) with respect to non-metastatic GEP-NETs. When the analyses were focused exclusively on distant metastases, five miRNAs resulted in being upregulated in metastatic tumors. Conversely, the selective analysis of only nodal metastases showed the presence of 21 upregulated and 11 downregulated miRNAs. Forty-four miRNAs were positively or negatively correlated with the Ki67 proliferation index. Comparison of miRNA expression profiles between metastatic cases and Ki67 revealed an overlap for three miRNAs, miR-150, miR-21, and miR-660, while comparison between primary tumors and metastases showed an overlap only in pancreatic (miR-127) and ileal cancers (let-7g, miR-200a and miR-331). In conclusion, no site-specific miRNA signature was demonstrated for different primary GEP-NETs, but some miRNAs demonstrated an association with tumor growth and, at the same time, with the presence of metastases, suggesting them as possible markers for cancer proliferation and spreading.

Panarelli et al. [50] compared miRNA expression profiles in four different types of GEP-NETs (pancreatic, ileal, appendiceal, and rectal NETs). By using an algorithm, authors identified miRNAs able to discriminate GEP-NETs based on their site of origin in the embryonic gut; expression levels of miR-615 and miR-92b allowed discrimination of midgut (ileum, appendix) from non-midgut (rectum, pancreas) NETs, expression levels of miR-125b; miR-192 and miR-149 discriminated ileal from appendix NETs; expression levels of miR-429 and miR-487b discriminated rectal from pancreatic NETs (pNETs). In addition, the expression levels of miR-328 discriminated low- and intermediate-grade pNETs. According to these findings, GEP-NETs could be potentially classified and graded by using specific miRNA signatures, coupled to morphological and immunohistochemistry-based histological evaluation.

Other studies focused the analysis of miRNA signatures on tissue-specific GEP-NETs.

Three studies investigated differentially expressed miRNAs which could be used as potential diagnostic and prognostic biomarkers in pNETs.

Grolmusz et al. [51] downloaded the miRNA expression profile of 40 pNETs from the Gene Expression Omnibus (GEO) database and, as a result, a total of 40 miRNAs were included in the discovery cohort for their reanalysis by using GeneSpring 12.6 software. In silico reanalysis showed 19 miRNAs with altered expression between different tumor grades. Authors selected five miRNAs (miR-21, miR-29a, miR-10a, miR-106b, and miR-101) for the subsequent validation in an independent larger cohort of pNETs. Expression levels of three of these miRNAs (miR-21, miR-10a, and miR-106b) were successfully confirmed as upregulated in more proliferative tumors. Comparison of expression levels of these three miRNAs in 18 metastatic vs. 41 non-metastatic patients showed that only miR-21 was significantly upregulated in patients with metastatic disease, while no differences in the expression levels of miR-10a and miR-106b were detected between the two groups of patients. Univariate analysis showed that higher tissue expression of miR-21, miR-10a, and miR-106b of primary pNETs were correlated with worse progression-free time and overall survival; however, multivariate analysis only confirmed miR-21 as an independent prognostic factor.

Similarly, Zhou et al. [52] processed data obtained from the GEO database, to explore the expression profile of miRNAs in pNETs and identify novel miRNA-mRNA regulatory network involved in the tumorigenesis. Authors evaluated the gene expression profile of six patients with pNETs compared with five healthy controls (HC). Bioinformatics and functional enrichment analysis revealed 28 differentially expressed miRNAs and 859 differentially expressed mRNAs, including 253 potential target genes, between these two groups according to *p*-value < 0.01 and |log2FC| ≥ 2. These target mRNAs were mainly enriched in the ABC transporters pathway. In this network, miR-7-2-3p, miR-429, miR-182-5p, miR-129-5p, and miR-148b-3p demonstrated the highest connectivity, while *KLF12*, *NFASC*, *PKIA*, and *RAB3B* were the mRNAs with the highest connectivity. As a result of PPI sub-network analysis, CXCL12 was the hub protein. They concluded that *KLF12* and *CXCL12*, genes involved in ABC transporters and type II-diabetes mellitus pathway, could affect the progression of pNETs, whereas further studies are required to demonstrate the role of these miRNAs in pNETs.

In the third study on pNETs, Gill et al. [53] compared the miRNA expression profiles in patients with pNETs, 14 with distant metastases and 23 with loco-regional metastases. Four miRNAs resulted significantly differentially expressed between the two groups of patients; miR-3653 was upregulated and miR-4417, miR-574-3p, and miR-664b-3p were downregulated in patients with distant metastases compared with the other group. Target prediction in silico analyses showed a potential pNET-related target only for miR-3653, the *ATRX* gene, involved in DNA methylation and chromatin remodeling. miR-3653 appears to be a suitable predictive biomarker candidate for invasion and distant metastases in pNETs.

The crosstalk between miRNAs and chromatin remodeling is suspected to be relevant in cancerogenic pathway. In this light, Klieser et al. [54] investigated a potential linkage between the expression levels of a specific subset of “proliferation-associated” miRNAs (miR-132-3p, miR-145-5p, miR-183-5p, miR-34a-5p, and miR-449a) and the expression of histone deacetylases (HDACs), as well as the clinical and biological features in 57 pNET samples. miRNAs ranked in descending order of expression levels were miR-145-5p, miR-34a-5p, miR-132-3p, miR-183-5p, and miR-449a. The correlation analysis between miRNA and HDAC expression patterns revealed that expression of miR-449a significantly correlated with expression of two members of HDAC family (HDAC3 and HDAC4). A linkage between miRNA expression levels and pNET clinical characteristics, such as tumor grading, proliferative activity, and hormone activity, was also found. The expression levels of miR-132-3p, miR-183-5p, and miR-34a-5p were associated with higher overall survival (OS) and disease-free survival (DFS values), whereas those of miR-449a were associated with reduced survival rate. Thus, miR-132-3p, miR-183-5p, and miR-34a-5p were considered “tumor suppressor miRNAs”, while miR-449 was regarded as “oncogenic miRNA”. Interestingly, the expression levels of miR-145-5p were associated with lower OS and enhanced DFS. Taken together, all these data demonstrated that specific miRNAs could be linked to HDAC expression in pNETs and, in particular, miR-449a could play a critical role in pNET proliferation and could be a potential prognostic factor for poor survival.

Two studies selectively analyzed miRNA expression in gastric neuroendocrine tumors (gNETs).

Dou et al. [55] assessed the differences in miRNA expression levels between type 1 gNET tissues and non-tumor gastric mucosal (NGM) tissues and their target genes, in order to discover the possible molecular mechanism of type 1 gNET recurrence. A microarray analysis was used to compare the miRNA expression profile between type 1 gNETs and NGM tissues, finding five downregulated miRNAs (miR-194-3p, miR-6752-3p, miR-6800-3p, miR-6889-3p, and miR-933) and one upregulated miRNA (miR-202-3p) in type 1 gNETs, according to the log_2_FC > 1.5 and *p*-value < 0.05. Authors chose to validate only the upregulated miR-202-3p; this qPCR-based analysis confirmed the upregulation of this miRNA in type 1 gNET tissues. Bioinformatic analysis identified the *DUSP1* gene, a tumor suppressor gene previously described in a variety of diseases and associated with gastric tumors, as a potential target of miR-202-3p. The in vitro cotransfection of 93T cells with the miR-202-3p mimic and wild-type 3′-UTR of *DUSP1* confirmed *DUSP1* as a direct target of miR-202-3p. In conclusion, this study evidenced the upregulation of miR-202-3p in type 1 gNET lesions and suggested it could play critical roles in the pathogenesis of these gastric tumors by repressing translation of *DUSP1* mRNA.

In the second study, Lloyd et al. [56] investigated whether gastrin may affect the expression of specific miRNAs, which in turn affect the expression of downstream proteins regulating key cellular processes involved in gastric tumor progression. First, miRNA PCR array analysis was carried out to identify differentially expressed miRNAs between human gastric adenocarcinoma cells stably transfected with the gastrin receptor CCK2R (AGS_GR_), treated with or without gastrin. Among the six differentially expressed miRNAs, only the expression levels of miR-376c and miR-222 were proved to be significant. Only miR-222 was selected for further qPCR validation because the expression level of miR-376c was below the threshold for detection by qPCR. Gastrin increased miR-222 expression levels in AGS_GR_ cells, with maximum changes observed at 10 nM G17 for 24 h. In vitro experiments revealed that the increase in miR-222 levels by gastrin occurred via its receptor and PKC and PI3K pathways in AGS_GR_ cells. Subsequently, the expression levels of this miRNA were assessed either in both blood and tissue of animal models or in both gastric corpus biopsies and serum samples from eight hypergastrinemic patients with autoimmune atrophic gastritis and type 1 gNETs. In both cases, miR-222 expression was observed to be increased, whereas it was decreased in patients following treatment with a CCK2R antagonist. Finally, in vitro and in vivo experiments showed that gastrin-induced miR-222 upregulation resulted in lower expression and cytoplasmic mislocalization of p27^kip1^, which in turn caused actin remodeling and increased migration in AGS_GR_ cells. Overall, these data demonstrated a novel mechanism that contributes to gastrin-induced gastric tumor development. Furthermore, miR-222 could be a potential biomarker of hypergastrinemia and of type 1 gNETs, as well as for monitoring response to treatment with CCK2R antagonists.

Finally, two studies investigated how miRNA expression profile correlates with GEP-NETs surgical or medical therapy, respectively, and if it could be related to different treatment outcomes.

Lee et al. [57] investigated miRNAs as potential prognostic biomarkers of pNETs in patients who underwent curative surgery. They first performed a NanoString nCounter analysis to compare miRNA expression levels between two primary pNET cases and their matched liver metastasis, identifying 18 differentially expressed miRNAs between the two groups. Among these 18, 8 miRNAs presenting a FC ≥ 2, were selected for further expression on independent samples (37 pNETs and their matched non-neoplastic pancreas tissues); an evaluation of correlation with clinical and biological features of the tumors was performed as well. Expression levels of miR-196a resulted to be increased in patients with advanced cancer stage or higher lymph node metastasis, American Joint Committee on Cancer stage II or higher, high mitotic index, high Ki67 labeling index, and recurrence. miR-142-5p expression was also significantly elevated in pNETs with high mitotic index, while no significant change in miR-27b levels was observed, although this miRNA was higher expressed in pNETs with high mitotic index. The ROC analysis was additionally performed to evaluate the potential of miR-196a, miR-27b, and miR-142-5p as prognostic biomarkers in predicting recurrence of resected pNET. This study demonstrated that tissue miR-196a could be a promising prognostic biomarker of pNET recurrence risk after surgery.

Bösch et al. [58] investigated the inter-individual miRNA expression profile before and after treatment with somatostatin analogs (SSA) in patients with GEP-NETs. In the first stage, expression profile of 758 human miRNAs was evaluated in eight GEP-NET patients, before and after administration of SSA therapy. Thirty-six differentially expressed miRNAs were identified. The validation step, performed by qPCR, on the same eight samples before and after therapy showed that the SSA treatment induced the upregulation of 3 miRNAs (let-7c-5p, miR-24-3p, and miR-215-5p) and the downregulation of 12 miRNAs (miR-10a-3p, miR-185-3p, miR-339-5p, miR-371a-5p, miR-4436b-5p, miR-4653-3p, miR-4793-3p, miR-619-5p, miR-1226-3p, miR-3137, miR-4455, and miR-4656). In addition, the individual pre- and post-treatment miRNA expression profiles were compared; let-7c-5p and miR-3137 resulted, respectively, as upregulated and downregulated in each patient after SSA therapy.

No studies have specifically analyzed miRNA expression in GEP-NET specimens from MEN1 patients.

Lu Y et al. [59] employed qPCR to validate 48 miRNAs resulted to be upregulated in pancreas cell lines treated with 25mM glucose. The authors showed that miR-17 promotes pancreatic beta cell proliferation through downregulation of menin in MIN6 cells. When treated with a high dose of glucose, these cells, an insulinoma-derived mouse pancreatic beta cell line, overexpressed miR-17, resulting in a dramatic promotion of cell proliferation presumably caused by the miR-17-driven inhibition of menin expression.

Table 2 summarizes information about differentially expressed miRNA in GEP-NETs.

## 4. miRNAs in Pituitary Tumors

Pituitary tumors can secrete various different hormones (functioning tumors) or be non-functioning adenomas. Some authors studied whether different miRNA expression patterns characterize different subtypes of secreting pituitary adenomas (PAs) and/or non-functioning tumors.

Müssnich et al. [60] analyzed differences in miRNA expression pattern between 12 growth hormone gonadotropin-secreting pituitary adenomas (G-PAs) and 3 normal pituitary (NP) samples, finding 44 upregulated miRNAs and 57 downregulated miRNAs in the gonadotroph samples, with an FC value higher than 2. Validation analysis of these differentially expressed miRNAs, by qPCR performed on a larger series of 21 samples of G-PAs (including those used for the microarray analysis), confirmed two miRNAs as downregulated (miR-432 and miR-410) and two as upregulated (miR-374b and miR-17) in tumors. miR-410 resulted to be the most downregulated in adenomas. Analysis of miR-410 expression was enlarged to 12 prolactin (PRL)-secreting pituitary adenomas (PRL-PAs) vs. 12 growth hormone (GH)-secreting pituitary adenomas (GH-PAs), showing that this miRNA was more expressed in almost all PRL-PAs and in half of the GH-PAs, thus suggesting that its downregulation is specific of gonadotropin-secreting adenomas. In silico analysis, with MiRwalk, miRanda, and TargetScan, revealed that miR-410 interacted with several mRNA targets, in particular the transcript of the *G2/mitotic-specific cyclin-B1* (*CCNB1*) gene, a member of G2/mitotic-specific cyclin-B1 previously reported to play a critical role in pituitary tumor development. To validate the effect of miR-410 on CCNB1 expression, authors analyzed CCNB1 protein levels by Western blot in HEK-293 cells transiently transfected with miR-410 or scrambled oligonucleotide, as well as the *CCNB1* mRNA expression in G-PA samples. miRNA-410 was shown to reduce both mRNA and protein levels of CCNB1. All these data indicated that reduction of miR-410 is selectively involved in the pathogenesis of G-PAs, and that restoring normal expression of this miRNA may represent a therapeutic approach to such pituitary tumors.

He et al. [61] analyzed how the miRNA expression profile differed between GH-PAs, PRL-PAs, non-functional pituitary adenomas (NF-PAs), and NP samples, by performing a Next-Generation Sequencing (NGS) analysis. They found 122, 70, and 30 miRNAs differentially expressed between GH-PAs, PRL-PAs, and NF-Pas, respectively, compared with NP tissues. The most dysregulated miRNAs were validated by qPCR on a series of 13 GH-PAs, 17 PRL-PAs, 42 NF-PAs, and 6 NP samples; expression levels of miR-34c-3p, miR-34b-5p, miR-338-5p, and miR-375 resulted in being significantly lower in the PRL-PA group compared with NP tissues. In the NF-PA group, miR-493-5p and miR-124-3p were significantly downregulated, while miR-181b-5p displayed the opposite trend. In GH-PA samples, miR-184 expression was significantly upregulated, while miR-124-3p expression was significantly downregulated. These data suggested these miRNAs as potential biomarkers for different types of PAs.

miR-410-3p has been showed to act as a possible tumor suppressor miRNA, thus, Grzywa et al. [62] studied its involvement in pituitary tumorigenesis by screening its expression in 75 with PAs (34 G-PAs, 30 GH-PAs, 5 adrenocorticotropin hormone (ACTH)-secreting pituitary adenomas (ACTH-PAs), 3 plurihormonal PAs, and 3 NF-PAs). miR-410-3p expression resulted to be different for each type of PA: Higher in GH-PAs, and lower in G-PAs and NF-PAs. In vitro experiments were carried out on G-PA-, GH-PA-, and ACTH-PA-derived cell lines, transfected with synthetic mimic of miR-410-3p or scrambled control, in order to elucidate the effect of this miRNA on the proliferation and invasion capacity. This miRNA significantly increased both the proliferation and invasiveness in cell lines derived from G-PAs and ACTH-PAs, as well as expression of CCNB1, and promoted the activation of MAPK, PTEN/AKT, and Signal Transducer and Activator of Transcription 3 (STAT3) signaling pathways. An opposite effect of miR-410-3p was, instead, demonstrated on GH-PA-derived cell lines. Results from this study seem to suggest that miR-410-3p acts as an oncomiR in G-PA and ACTH-PA cells or as a tumor suppressor miRNA in GH-PA cells.

Zhen et al. [63] explored the role of miR-524-5p, whose decreased expression was previously reported in NF-PAs compared to NP tissues, as a tumor suppressor in PAs. Twenty pituitary-derived folliculostellate (PDFS) primary cell lines, isolated from NF-PA patients, were stably transfected with recombinant lentivirus containing human miR-524-5p. The induced overexpression of miR-524-5p suppressed PDFS cell proliferation, clonogenicity, and tumorigenicity, and inhibited their migration and invasion ability. Moreover, the overexpression of this miRNA downregulated the expression of the pituitary tumor-transforming gene 1 binding factor (*PBF/PTTG1IP*) at both mRNA and protein levels. Taken together, these findings suggest a therapeutic potential of miR-524-5p in NF-PAs.

Most pituitary tumors are noncancerous adenomas. However, some of them are characterized by excessive growth (macroadenomas) that can put pressure on or invade the normal pituitary gland and nearby structures. Some studies investigated if some specific miRNAs can be associated with a higher PA proliferation rate, a major tumor invasiveness and/or aggressiveness. They are briefly described below.

miR-132 and miR- 15a/16 clusters have been previously reported to be implicated in the pathogenesis of many types of cancer. Renjie et al. [64] assessed the expression levels of these miRNAs in tissues of invasive and non-invasive pituitary tumors isolated from 16 patients diagnosed with PAs. The expression levels of these miRNAs were significantly lower in both invasive pituitary tumor tissues and their derived cell lines, compared with non-invasive tumor tissues. In vitro analyses were performed to investigate the molecular mechanism of the regulation of miR-132 and miR-15a/16 on cell proliferation, invasion, migration, and on epithelial to mesenchymal transition (EMT). The overexpression of these miRNAs was shown to promote the suppression of pituitary tumor cell proliferation, migration, and invasion, respectively, as well as inhibiting the expression of proteins involved in EMT (i.e., Twist, vimentin, and N-cadherin), in a mechanism sex determining region Y-box protein 5 (*Sox5*)-mediated, revealing these miRNAs as potential selective therapeutic targets for invasive pituitary tumor.

Wu et al. [65] compared the expression profile of 2006 human miRNAs between six invasive and six non-invasive NF-PAs. Six miRNAs resulted to be differentially expressed, according to the FC (≥2 or <0.5) and *p*-value (<0.05) values. In particular, four miRNAs (miR-181b-5p, miR-181d, miR-191-3p, and miR-598) were upregulated in the invasive group compared with the non-invasive one, while two miRNAs (miR-3676-5p and miR-383) were downregulated. In order to further understand the function and the related signaling pathways of these miRNAs, enrichment analysis of Gene Ontology (GO) and Kyoto Encyclopedia of Genes and Genomes (KEGG) annotation tools were performed. The authors found that these differentially expressed miRNAs were potentially involved in many signal transduction pathways, including PRL signal transduction pathways, endocrine and other factors regulating calcium reabsorption, fatty acid metabolism, neurotransmitter-ligand interaction by endocrine function, etc. Finally, they selected three miRNAs (miR-181b-5p, miR-181d, and miR-191-3p) for a further validation on an independent series of four non-invasive NF-PAs and four invasive NF-PAs. They chose miR-181a-5p instead of miR-181b-5p and miR-181d to verify the results from microarray analysis since they only had miR-181a-5p in the miRNA probe library, and because this miRNA is produced by the same precursor and is highly isogenic to the sequences of miR-181b-5p and miR-181d. The expression levels of miR-181a-5p and miR-191-3p were significantly upregulated in invasive pituitary tumors, in accordance with the microarray results. They concluded that these miRNAs could be potential diagnostic and therapeutic biomarkers for invasive tumors.

The purpose of a study by He et al. [66] was to investigate the role of miR-148b-3p, miR-152/activated leukocyte antigen molecule (ALCAM) axis in human PAs. Expression levels of miR-148b and miR-152 were analyzed in 10 invasive PA (IPA) and 10 non-invasive PA (non-IPA) samples. A decreased expression of both these miRNAs was observed in IPA samples. The role of these two molecules on the proliferation, invasion, and apoptosis was studied in vitro by using human PA cells transfected with miR-148b-3p and miR-152 mimics, ALCAM mimic, and siRNA, finding that the overexpression of miR-148b-3p and miR-152 inhibits proliferation and invasion, and promotes apoptosis, probably via inhibiting ALCAM expression at both mRNA and protein levels. Further investigations are needed to confirm the role of the miR-148b-3p, miR-152/ALCAM axis in PA tumorigenesis and as potential diagnostic tools and molecular targets for PA treatment.

miR-543 has been previously shown to play a role as an oncogene in different types of cancers. For this reason, Shen et al. [67] selected this putative oncomiR and investigated its role in PA development. First, they profiled a qPCR assay to explore the correlation between miR-543 expression and PA progression in 66 IPA and 71 non-IPA tissues, finding that miR-543 was significantly upregulated in the IPA group. By in silico analysis, reporter gene assay, and Western blot experiments, they observed that the overexpression of miR-543 could regulate the Wnt/β-catenin pathway by specifically binding to Smad7. Moreover, expression studies performed in HP75 cells transfected with miR-543, negative control (NC) mimic, miR-543 inhibitor and NC inhibitor, respectively, showed that the overexpression of miR-543 promoted cell proliferation, migration and invasion, and decreased cell apoptosis through the activation of Wnt/β-catenin signaling pathway by targeting *Smad7*. According to the authors, miR-543, Smad7, and Wnt/β-catenin signaling pathway could be an alternative method for the currently existing therapeutic approaches for PA, even though further investigations are required to illustrate the efficacy and safety of the use of these molecules as potential therapeutic methods in the treatment of PA.

Wang et al. [68] chose miR-133, an miRNA that was reported as a tumor suppressor in different types of cancer metastasis, to investigate its possible role, and the underlying molecular mechanisms, in pituitary tumor cell migration and invasive capacity. The use of miRBase and TargetScan software identified *FOXC1* as a potential target gene of this miRNA. To verify this result in vitro, a dual luciferase reporter assay was performed for the analysis of FOXC1 mRNA and encoded protein levels in PA cell lines transfected with miR-133a/b mimics. The authors showed an inverse correlation between miR-133 and *FOXC1* expression, confirming that FOXC1 undergoes a direct negative control by this miRNA. Cell migration and invasion assays revealed that miR-133 can inhibit both these processes in tumor cells. Western blot analysis and EMT process monitoring showed that the upregulation of miR-133 increased the expression of EMT-related proteins and favored the EMT process, while the overexpression of *FOXC1* had opposite effects. The qPCR analysis showed that expression of miR-133 was upregulated, while the expression of FOCX1 was downregulated in tumor tissues compared with the corresponding adjacent normal samples. Results from this study indicated miR-133 as a potential therapeutic target for the treatment of IPAs.

Given that miR-26a and its target, the *pleomorphic adenoma gene 1* (*PLAG1*), have been reported to be abnormally expressed in PAs, the aim of a study by Yu et al. [69] was to investigate the relationship between these molecules and the invasiveness of pituitary tumors. Expression levels of miR-26a and PLAG1 have been assayed in 70 PA samples (31 IPAs and 39 non-IPAs) with respect to NP. miR-26a expression was significantly higher in pituitary tumor tissues, while *PLAG1* mRNA expression was remarkably downregulated in adenomas, especially in IPA tumors. Western blotting analysis showed that NP tissues had higher PLAG1 protein levels compared to non-IPA tissues, and that the latter had higher PLAG1 protein compared to IPA tissues. Patients with miR-26 overexpression and *PLAG1* downregulation showed unfavorable prognosis, according to the Cox regression model. ROC analysis revealed that miR-26a had the highest AUC values in discriminating IPAs from non-IPAs (AUC = 0.889), compared with PLAG1 (AUC= 0.818). In summary, authors assumed that miR-26a could play a critical role both in the occurrence and invasiveness of PAs, probably via inhibiting *PLAG1* expression. Therefore, miR-26a and PLAG1 could represent possible molecular targets for PA therapy.

The aim of a study by Yu et al. [70] was to investigate the relationship between four miRNAs (miR-24, miR-93, miR-126, and miR-34a), chosen based on experiments, in silico software, and the literature, and proteins related to the pituitary tumor invasion mechanism (i.e., basic fibroblast growth factor 2 (FGF2), pituitary tumor transforming gene (PTTG), CCNB1, survivin, focal adhesion kinase (FAK), and microvessel density (MVD)). The aforementioned four miRNAs were chosen according to the following two criteria: (1) Obtained by at least three miRNA-target-prediction algorithms, or demonstrated, in vitro, to directly target the mRNA and inhibit the expression of one of the studied proteins; (2) involved in the invasiveness of other tumors and involved in the pathogenesis of PAs with a still unclear involvement in PA invasiveness. Expression of these four miRNAs was assessed in 30 IPA vs. 30 non-IPA cases. All FGF2, FAK, PTTG, CCNB1, and MVD proteins were overexpressed in the invasive group compared with the non-invasive group, while the expression levels of miR-24, miR-34a, and miR-93 displayed an opposite trend. No significant difference in miR-126 expression was found among the two groups of PAs. This study demonstrated a direct inverse relationship between the expression levels of miR-24, miR-34a, and miR-93 and invasiveness-related proteins during the PA invasion process, indicating these molecules as potential molecular targets for anti-invasive therapies of PAs.

Wang et al. [71] sought to identify differentially expressed miRNAs between aggressive and non-aggressive PRL-PAs using microarray data of the GEO database. They only selected the GSE46294 dataset, which included four aggressive PRL-PA samples and eight non-aggressive PRL-PA samples. Bioinformatic analysis found 43 differentially expressed miRNAs (19 upregulated and 24 downregulated) between these two tumor types. Among these, let-7d*, miR-138-1*, and miR-489 resulted in being the three most highly upregulated miRNAs, while miR-520b, miR-671-3p, and miR-875-5p were the three most highly downregulated miRNAs, based on their FC values. According to the results from miRTarBase, the three most highly upregulated and downregulated miRNAs were predicted to target 170 and 680 potential genes, respectively. Functional and pathway enrichment analyses showed that these target genes were involved in the regulation of transcription from RNA polymerase II promoter, DNA-templated transcription, transcription factor activity, protein binding, and Wnt signaling pathway. Among the total of these 850 target genes, authors selected the 10 genes with the highest degree of connectivity with the three upregulated and three downregulated differentially expressed miRNAs, and found that the majority of these 10 genes were potentially regulated by miR-489 and miR-520b, suggesting these two miRNAs as potential markers for the diagnosis and treatment of aggressive PRL-PAs.

García-Martínez et al. [72] elucidated the relationship of E2F1 and miR-17-92 cluster (miR-17-5p and miR-20a), previously found as deregulated in cancer, with the invasiveness and proliferation of different kinds of pituitary tumors. They compared the expression levels of E2F1, MYC, pri-miR-17~92, miR-17-5p, and miR-20a between 9 NP tissues and 60 PAs (29 G-PAs, 15 functioning GH-PAs, and 16 ACTH-PAs of which 8 were silent). After establishing invasiveness and proliferation according to the Knosp classification and to molecular expression of Ki67 ≥2.59, respectively, they found that E2F1 expression was higher in invasive tumors compared with non-invasive ones and GH-PAs. Moreover, E2F1 was overexpressed in the G-PAs and the eight silent ACTH-PAs, and normally expressed in the functioning ones (GH-PA adenomas and the eight secreting ACTH-PAs). MYC also showed higher expression in invasive GH-PAs than in non-invasive ones, but not in the whole series. Indeed, the expression of MYC was lower in invasive than in non-invasive G-PAs. Unlike E2F1 and MYC, no differences were observed among the expression of pri-miR-17-92 and mature miRNA and invasiveness behavior in the whole series or by subtypes. miR-17-5p expression levels were significantly more expressed in proliferative than in non-proliferative tumors in the whole series but no differences were observed in the subtypes. On the contrary, there was no correlation in the expression of E2F1 or MYC among the three categories of pituitary tumors, both in the whole series or by cancer subtypes. Overall, they assumed that E2F1 and miR-17-5p could be promising good biomarkers of invasiveness and proliferation, respectively, thus helping the clinical management of pituitary tumors.

The phosphatase and tensin homolog deleted on chromosome 10 (PTEN)-phosphatidylinositol 3-kinases (PI3K)/AKT signaling pathway is known to play a critical role in tumor cell migration and invasion processes. Two studies investigated its involvement in pituitary tumorigenesis and tumor progression in relationship with miR-106b.

In 2016, Zhou et al. [73] analyzed the expression of miR-106b and PTEN in PAs and NP in order to elucidate whether this miRNA promotes proliferation and invasion of pituitary tumor cells via the PI3K/AKT signaling pathway by regulating *PTEN* expression. Expression levels of miR-106b and *PTEN* mRNA were compared between 29 cases of IPA, 26 cases of non-IPAs, and 8 NPs. Expression of miR-106b resulted in being significantly higher in IPA tissues compared with non-IPA and NP tissues, while no significant different expression was observed between non-IPA and NP tissues. qPCR and Western blot assays revealed that the downregulation of miR-106b in EC-1 or EC9706 cells resulted in an increase of PTEN expression both at mRNA and protein levels, indicating that PTEN is a direct target of miR-106b. In vitro analysis in AtT-20 cells, a mouse ACTH-PA-derived cell line, transfected with miR-106b, mimics miR-106b inhibitor, PTEN expression plasmid, and miR-106b mimics + PTEN expression plasmid, respectively, showed that miR-106b promoted AtT-20 cell proliferation and invasion through the PI3K/AKT pathway by negatively targeting PTEN.

One year later, Zheng et al. [74] studied the relationship between the miR-106/PTEN-PI3K/AKT signal pathway and invasiveness of PAs. qPCR results showed that miR-106b expression level in 50 PA patients was significantly increased compared to that in 10 NP tissues, while *PTEN* mRNA were remarkably depressed. In PA patients, miR-106b expression was higher in 32 invasive tumors compared with 18 non-IPAs, while *PTEN* mRNA showed an opposite pattern of expression. Following proliferation, migration, and invasion assays performed on HP75 cells transfected with miR-106b inhibitors or lipofectamine (negative control), authors found that the inhibition of miR-106b remarkably suppressed proliferation and anchorage-independent growth of HP75 cells, significantly decreased invasion ability of cells, and increased PTEN expression, thus affecting the activity of downstream PI3K/AKT signaling pathway.

Overall, results from these two studies suggest that miR-106b and PTEN could be potential diagnostic biomarkers or therapeutic targets for PA treatment and for prevention of tumor dissemination.

The same year, Garbicz et al. [75] investigated the expression pattern of the miR-106b~25 cluster (which includes miR-25-3p, miR-93-3p, miR-93-5p, and miR-106b-5p) and its host gene, the minichromosome maintenance complex component 7 (*MCM7*) in a cohort of 25 patients with Cushing’s disease and ACTH-PA (composed of 5 patients with Crooke’s cell adenoma (CCA), 13 patients with IPA, and 9 patients with non-IPA). All miRNAs belonging to the miR-106b~25 cluster were upregulated in the CCA group with respect to the IPA group, while only miR-93-5p expression levels were significantly higher in invasive compared to non-invasive tumors. Results from immunohistochemistry analysis showed a significant increase in MCM7 and Ki67 labeling indices in invasive ACTH-PAs. ROC analysis revealed that the combination of MCM7 LI and miR-106b~25 cluster expression had the highest significant AUC values in distinguishing invasive from non-invasive tumors, and also had a significant discriminatory ability to predict post-operative tumor recurrence/progression. The authors concluded that miR-106b~25 and its host gene *MCM7* are potential novel biomarkers for invasive ACTH-PAs.

All the above reported studies analyzed miRNA expression in sporadic non-MEN1 pituitary tumors.

Concerning MEN1-related PAs, a recent study by Lines KE et al. [76] investigated the expression of miR-15a, miR-16-1, and let-7a in pituitary tumors from a mouse model of MEN1 tumorigenesis (i.e., Men1^+/−^ mice), finding all three miRNAs to be significantly downregulated in MEN1 PAs compared to normal wild type pituitaries. In addition, miR-15a and miR-16-1 expression inversely correlated with expression of CCD1, presumably being a trigger of loss of cell cycle control and pituitary tumorigenesis. Additional studies of *Men1* knock-out in the AtT20 mouse pituitary cell line resulted in significantly decreased expression of miR-15a, as a direct result of loss of wild type menin expression. Restoring miR-15a and miR-16-1 normal expression could be a therapeutic molecular approach to block pituitary tumorigenesis in *MEN1* mutated patients.

Table 3 summarizes information about differentially expressed miRNAs in pituitary tumors.

## 5. miR-24 and Menin

Currently, miR-24 is the only miRNA that has been associated with menin expression regulation in more than one study. It is directly involved in MEN1 tumorigenesis.

As described above, Luzi et al. [26] demonstrated a direct regulative feedback-loop between miR-24-1 and menin, showing that miR-24 is a negative regulator of menin expression in PAs from MEN1 patients without somatic LOH in tumor tissues, acting as an epigenetic trigger of tumorigenesis.

Two years later, Vijayaraghavan J et al. [77] showed that miR-24 directly decreases menin levels and impacts downstream cell cycle inhibitors in MIN6 insulinoma-derived mouse pancreatic beta cell line and in Blox5 human immortalized beta cell line. The authors confirmed the existence of a feedback regulation between miR-24 and menin, also in pancreatic islets, hypothesizing that miR-24 could have a key role in MEN1 tumorigenesis of endocrine pancreas, but could also be involved in pancreatic beta cell function and development.

Liver is not a primary target tissue of MEN1 tumorigenesis. Nevertheless, Ehrlich L et al. [78] confirmed that miR-24 negatively regulates menin expression in the liver, through in vitro expression evaluations in intrahepatic and extrahepatic cholangiocarcinoma cell lines (Mz-ChA-1, TFK-1, SG231, CCLP, HuCCT-1, and HuH-28) and a nonmalignant human intrahepatic biliary line (H69), demonstrating that miR-24 inhibition decreases cholangiocarcinoma proliferation via increasing menin expression.

The effects of miR-24 on the biological behavior of lung cancer cells was studied via transfection of a human lung carcinoma cell line (A549) and a human lung small cell cancer-derived cell line (NCI-H446) with miR-24 mimic or miR-24 inhibitor, with respect to null transfected controls [79]. Overexpression of miR-24 was found to inhibit menin expression, affecting the activity of SMAD3, in lung cancers. Authors speculated that miR-24 might promote cell growth and metastasis and inhibit apoptosis of lung cells, by directly targeting *MEN1* mRNA, being responsible for both tumor development and malignant progression.

**Figure 1:**
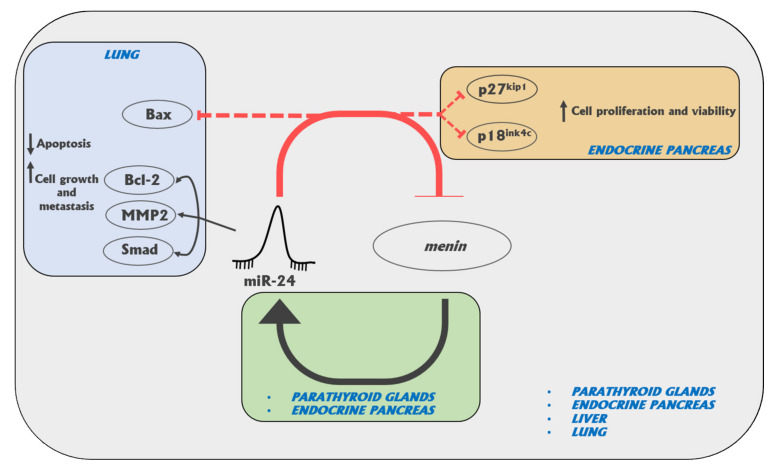
Diagram of the possible targets of miR-24 in canonical and non-canonical MEN1-affected tissues [26,77,78,79].

In conclusion, miR-24 can be considered as an oncomir, whose overexpression increases cell growth and favors tumor development via the inhibition of menin oncosuppressor activity, not only in the canonical MEN1-affected neuroendocrine tissues (Figure 1). Currently, this miRNA appears as the most promising target for molecular therapy, aimed to block MEN1 tumorigenesis, by inhibiting its overexpression in MEN1 neuroendocrine target tissues before the occurrence of somatic loss of the second wild type copy of the *MEN1* gene.

## 6. c-miRNAs in MEN1 Neuroendocrine Target Tissues

Li et al. [80] investigated the serum expression levels of nine miRNAs, previously reported to be deregulated at tissue level, (miR-31, miR-96, miR-129-5p, miR-133a, miR-182, miR-183, miR-196a, miR-200a, and miR-215) between two distinct cohorts of oncologic patients (the first cohort consisted of 21 untreated patients with small intestinal NETs (siNETs) (7 primary tumors (PTs), 7 with lymph node metastases (LNMs), 7 with liver metastases (LMs)); the second cohort consisted of 21 SSA-treated patients with siNETs (7 SSA-PTs, 7 SSA-LNMs, and 7 SSA-LMs)), both with respect to 7 HC. In the first cohort, the serum expression levels of miR-96 was significantly higher at the stages of PT and LM, while those of miR-182, miR-196a and miR-200a were significantly higher only in LM patients vs. HC. In addition, the expression levels of miR-31, miR-129-5p, miR-133a, and miR-215 were significantly downregulated in siNET patients at all different tumor stages compared with HC. In the second cohort, the levels of miR-96, miR-182, miR-183, miR-196a, and miR-200a were significantly higher in SSA-treated patients at all different tumor stages compared with HC, while the levels of miR-31, miR-129-5p, miR-133a, and miR-215 were significantly lower in SSA-treated patients at all different tumor stages compared to HC. By comparing serum miRNA levels in untreated and SSA-treated siNETs, expression of miR-96, miR-182, miR-183, miR-196a, and miR-200a resulted to be lower in siNET untreated patients compared to SSA-treated patients at all different tumor stages, while no significant difference in the expression levels of miR-31, mi-129-5p, miR-133a, and miR-215 was observed between the two groups of patients. Overall, these findings indicate that these c-miRNAs could be serum markers of drug response, non-invasive for the patient, to be routinely dosed during therapy.

Xu et al. [81] investigated whether exosome miRNAs isolated from conditioned medium of human pancreas tumor cell lines (PANC-1, MIA-PaCa-2, BxPC-3), the human pancreas nestin expressing cell line (hTERT-HPNE) as representative of healthy pancreas, and from plasma samples of 15 patients with primary pancreas tumors and 15 HC, could be associated with localized pancreatic cancer. First, they profiled miRNA levels in PANC-1 and hTERT-HPNE exosomes. Among the top 30 differentially expressed miRNAs between the two cell lines, authors selected miR-196a, miR-196b, and miR-1246, because of their selective expression and high abundance in PANC-1 exosomes. Expression of these three miRNAs were furtherly analyzed on exosomes derived from PANC-1, MIA-PaCa-2, and BxPC-3 tumor cells vs. hTERT-HPNE control cell line, showing significantly higher expression levels of all those three miRNAs in exosomes derived from cancer cells. Expression levels of miR-1246, miR-196a, and miR-196b were then analyzed in plasma exosomes of patients with localized pancreatic tumors compared with HC, showing increased levels of miR-196a and miR-1246 in subjects with cancer; the ROC analysis confirmed diagnostic value of these two miRNAs in predicting localized pancreatic cancer. No significant difference was observed in plasma exosome miR-196b expression. A subsequent analysis of the localized pancreatic cancer subtypes showed plasma exosome miR-196a as more expressed in pancreatic ductal adenocarcinoma (PDAC), and plasma exosome miR-1246 significantly higher in patients with intraductal papillary mucinous neoplasms (IPMN). Conversely, there were no differences in the plasma exosome miR-196a and miR-1246 expression levels between NET patients and HC. In conclusion, miR-196a and miR1246 could be potential biomarkers for diagnosis of PDAC and IPMN, respectively.

Heverhagen et al. [82] sought to identify serum miRNAs as potential noninvasive biomarkers in siNETs. In the first phase, they analyzed a set of 84 miRNAs in tissue samples of 15 patients with primary siNETs vs. 7 HC samples. Twenty-six miRNAs were differentially expressed in tumor tissue samples compared to controls. The authors selected 12 downregulated miRNAs (miR-9-5p, miR-124-3p, miR-143-3p, miR-144-3p, miR-122-5p, miR-100-5p, miR-130a-3p, miR-28-5p, miR-223-3p, miR-21-5p, miR-126-3p, and miR-142-3p) and 2 upregulated miRNAs (miR-7-5p and miR-96-5p) for further analysis. These miRNAs were validated in 15 additional tissue samples from both primary tumor and liver metastases sources. Since miR-7-5p showed a significant upregulation in tumors compared to healthy tissue, its expression was further analyzed in the serum of 32 patients with siNETs vs. 25 HC. As a result of this analysis, the expression levels of miR-7-5p were significantly upregulated in serum samples of siNET patients compared with HC; the ROC analysis confirmed its diagnostic potential in discriminating patients with or without siNETs. Overall, these findings revealed miR-7-5p as potential serum biomarker for siNETs. A validation of these preliminary data would be required in large-scale prospective studies.

Lu et al. [83] analyzed the expression profile of miR-16 in the serum of 36 patients with pituitary tumor compared with 8 HC. A decrease of miR-16 expression was observed in the serum of pituitary tumor patients compared with HC. Furthermore, its elevated expression correlated with longer survival compared to low expression of miR-16 in pituitary tumor patients. Based on the results derived from cell models, transiently transfected with miR-16 mimics and NC, the authors observed that the treatment with miR-16 mimics decreased cell proliferation and induced apoptosis of HP75 cells in a dose-dependent manner, compared to NC, and in particular, miR-16 overexpression suppressed phosphorylation-(p)-p38, NF-κB, MMP-9, and VEGFR2 protein expression and induced p27, Bax protein expression, and caspase-3/8 activities in HP75 cells. Subsequently, VEGFR2-silenced and NF-κB-silenced cell models were used to study the mechanisms by which miR-16 affected proliferation, apoptosis, and angiogenesis of pituitary tumors. They observed that VEGFR2 or NF-κB suppression reduced the effects of miR-16 overexpression on p-p38, NF-κB, MMP-9, and VEGFR2 protein expression inhibition in HP75 cells. Taken together, these findings demonstrated that miR-16 expression suppressed cell proliferation, induced apoptosis and reduced angiogenesis of pituitary cancer through the VEGFR2/p38/NF-κB signaling pathway, thus suggesting miR-16 as a potential therapeutic target for clinical management of pituitary tumors.

Németh et al. [84] evaluated miRNA expression profile in the plasma of patients with PAs. In the discovery phase, they evaluated differentially expressed miRNAs in the plasma of 36 paired pre-operative and post-operative samples obtained from patients with different types of PAs (10-10 from patients with G-PAs, 4-4 from GH-PAs, and 4-4 from NF-PAs) compared with 2 HC. Fifteen differentially expressed miRNAs were identified between different adenoma subtypes, while 14 miRNAs were differentially expressed between healthy and patient groups. Furthermore, in order to avoid perioperative effects on the expression of miRNAs, the authors compared miRNA expression profile in the plasma of pre-operative and late post-operative samples, grouped by different histological types, by identifying 3, 7, and 66 differentially expressed miRNAs between the two groups pre- and post-operative plasma samples in GH-PA, G-PA, and NF-PA groups, respectively. Individual qPCR assays were used to validate NGS results on a greater population. Only miR‒143-3p levels were significantly downregulated in post-operative G-PAs in the validation phase. When comparing its expression in preoperative plasma samples from the different histological groups, a significantly higher expression was found in G-PA samples. The ROC analysis confirmed the diagnostic potential of miR-143-3p in discriminating between plasma samples obtained from pre-operative and late post-operative patients with G-PAs (AUC = 0.79). Their results confirmed that a general low level of c-miRNAs in plasma from patients compared with HC, and especially miR‒143-3p, could be a potential biomarker for patient follow-up only for G-PAs.

Zhao et al. [85] studied the difference of serum exosomal-derived miRNA expression profile between six GH-PA patients and six HC, to explore c-miRNAs as potential biomarkers in GH-PAs. From the exosomal miRNA expression profiling, they identified 169 differently expressed miRNAs, including 121 upregulated miRNAs and 48 downregulated miRNAs, in GH-PA samples compared with Hs, according to the FC (>2) and *p*-values (<0.05). Among the 169 differentially expressed miRNAs, only two miRNAs (miR-423-5p and miR-320a) were validated to be lower expressed in GH-PAs, by using miRSCan Panel ChipTM qPCR. Transcriptomics and proteomics analysis indicated that expression of *PTTG1* and *SYT1* genes was inhibited both at the mRNA and protein levels by miR-423-5p and was higher in GH-PAs compared with NP samples. In vitro experiments showed that miR-423-5p induced cell apoptosis, inhibited cell proliferation, and reduced GH release and migration in cell models transfected with miR-423-5p mimics. Based on these findings, the serum exosomal miRNAs, and, in particular, miR-423-5p, were important for GH3 cell proliferation and could promote tumorigenesis in GH-PAs and could therefore be potential diagnostic biomarkers for such diseases.

No specific studies are currently available on c-miRNA in MEN1 tumors.

Table 4 summarizes information about c-miRNAs as candidate biomarkers in sporadic non-MEN1 associated tumors.

## 7. Discussion

Despite recent advances in diagnostic strategies and treatment options for MEN1-associated tumors, patients with MEN1 continue to have a reduced life expectancy with an average age of death around 55–60 years [86]. Delayed diagnosis has been associated with increased mobility and mortality. The latest Clinical Practice Guidelines for MEN1, published in 2012 by the Endocrine Society, recommended that patients with MEN1-associated tumors and asymptomatic first-degree relatives should be subjected to intensive, life-long, clinical, biochemical, and radiological screening for the earliest possible diagnosis of MEN1-associated tumors [87].

Since the identification of the *MEN1* gene in 1997, genetic screening has been demonstrated to be fundamental in clinical practice, able to anticipate the diagnosis of MEN1 in asymptomatic carriers more than 10 years before the appearance of biochemical signs of the disease. Unfortunately, no genotype/phenotype correlation exists, preventing the ability to foresee MEN1 individual clinical phenotype and, consequently, reducing the possibility of a personalized diagnostic and therapeutic plan for MEN1-associated tumors and related endocrine syndromes.

Recently, it has been suggested that epigenetic mechanisms could affect the disease phenotype in patients carrying the same *MEN1* gene mutation [1]. Among them, miRNAs have shown increasing evidence of a direct role in human tumorigenesis, both for sporadic and hereditary cancers. Deregulation of specific miRNAs cause tumor development, malignant progression, and metastases, suggesting the possibility of using these molecules both as diagnostic and prognostic tools of tumor grading and severity, and possible targets for innovative molecular anti-cancer therapies.

As described in this review, several deregulated miRNAs have been associated with the sporadic tumorigenesis affecting the three main neuroendocrine target tissues of MEN1 syndrome. On the other hand, very few studies of miRNA expression analysis in tumor specimens from MEN1 patients have been conducted.

Currently published results strongly indicate miR-24 as a key pro-oncogenic factor, targeting MEN1 mRNA, both in MEN1 neoplasms and other non-MEN1 sporadic tumors, indicating it as a promising target for an anti-cancer therapy, aimed to restore the expression of menin.

Recently, experimental evidence has shown the potential use of antisense oligonucleotides to suppress aberrant miRNA function (AMOs) in several human pathologies, and hence the design of specific AMO-based strategies could find future application as RNA-based therapy that could prevent or at least to delay the onset of MEN 1-associated tumors [88].

In addition to intracellular miRNAs, c-miRNAs have been demonstrated to be potential biomarkers in various human malignancies, useful for the diagnosis of tumors together with conventional diagnostic approaches, as prognostic factors and easily detected markers of response to anti-cancer therapies. Indeed, mature miRNAs can be released in different extracellular biofluids. The expression patterns in biological fluids of these highly stable and quickly detectable circulating molecules are strongly dependent on physiological and pathological conditions [33,34,39,40]. In recent years, several studies have shown that c-miRNAs are potential non-invasive biomarkers in various diseases, including cancer [41].

Based on the current review, only a few studies have evaluated c-miRNA expression in biological fluids derived from patients affected by tumors in tissues in whom MEN1 syndrome has a higher penetrance, such as pituitary gland and GEP tract tumors (Table 4). The identification of a specific c-miRNA signature associated with the different clinical phenotypes of MEN1 could permit the development of effective and non-invasive diagnostic tools for the assessment of MEN1 status, which, in addition to the pre-existing ones, could allow more accurate and earlier diagnosis of MEN1 and its clinical manifestations. This would provide the possibility to direct patients towards an individual therapeutic program, greatly benefiting from early identification at a presymptomatic stage. In fact, experimental evidence has shown that c-miRNA expression profiles could facilitate the correct classification of different cancer subtypes and grades of differentiation [43]. Despite an increasing interest of c-miRNAs as promising biomarkers in different types of diseases, the scientific community is not that close to identifying a miRNA signature either, because there are only a few studies and because those available in literature have suggested different c-miRNAs as possible candidate biomarkers in the three main MEN1-associated endocrinopathies.

In addition, several issues must be overcome before the use of these small molecules as disease-specific biomarkers can be put into clinical practice. In fact, it has been observed that c-miRNA expression profiles can be affected by inter- and intra-individual differences, such as ethnicity [89], gender [90,91], as well as external factors and life-style, such as drug assumption [92], smoking habits [93], diet [94,95], and physical activity [96]. Furthermore, it is well known that differences in starting material, RNA extraction yield, and reaction efficiency should be taken into account for the measurement of miRNAs in biological fluids. In this regard, one of the major challenges for c-miRNA expression analysis is the suitable endogenous miRNAs for data normalization [97]. Currently, no consensus exists regarding the optimal single or set of reference genes to be used for normalizing miRNA expression data, thus hindering the comparison of results obtained from different studies.

Therefore, a possible way forward before their clinical application could be the identification of a set of c-miRNAs by using large-scale set techniques for massive miRNAs profiling, which do not rule out c-miRNAs a priori. After identifying potential miRNA biomarkers through these methodologies, these candidate molecules should be validated on a much larger independent cohort in order to determine their potential diagnostic and prognostic application in MEN1 syndrome.

In conclusion, this review provides a general overview of the candidate miRNAs in MEN1 tumor development that could be possible targets for the future development of RNA antagomirs-based strategies to control MEN1 tumorigenesis. In perspective, future experiments, aimed both at increasing knowledge about the molecular and cellular mechanisms that determine the onset and progression of MEN1, and at demonstrating the potential application of c-miRNAs as specific and sensitive biomarkers for the assessment of MEN1 status, are required to confirm the data obtained to date, and to translate results of basic research to clinical practice.

## Figures and Tables

**Table 1:** Overview of differentially expressed microRNAs (miRNA) in parathyroid glands.

Tissues/Cells	miRNAs	Potential Biological Role in Parathyroid Glands	Platform for miRNA Expression Analysis	Internal Control for qPCR	AUC Value_ROC	Study
PTC (15), 19 atypical PTA (19), PTA (46), NPT (6)	miR-372 (↑)	Role in parathyroid tumorigenesis	qPCR	HMBS and B2M	/	[44]
PTC (7), sporadic PTA (41)	miR-222 (↑), miR-139 (↓), miR-30b (↓), miR-517c (↓), miR-126 * (↓)	/	qPCR	U6	0.864 (miR-30b) 0.747 (miR-139) 0.888 (miR-30b + miR-139)	[45]
Sporadic PTA (28), hereditary PTA (15), NPT (27)	miR-199b-5p (↑ o ↓)	/	Microarray, qPCR	RNU6	0.863 (miR-199b-5p)	[46]
MEN1 PTA (8), sporadic non-MEN1 PTA (3), NPT (1)	miR-24-1 (↑)	Role in MEN1 parathyroid tumorigenesis	Northern blot, qPCR	5S RNA, 18S RNA	/	[26]
LOH-MEN1 (4), non-LOH MEN1 (3), sporadic parathyroid adenomas (2)	miR-4258 (↑ o ↓), miR-664 (↑ o ↓), miR-1301 (↑)	Role in parathyroid tumorigenesis	Microarray, qPCR	5S RNA	/	[27]
MEN1-associated PHPT (16), sporadic PHPT (40)	miR-24 (↑), miR-28 (↑)	Significance in PHPT pathogenesis	qPCR	RNU6B	/	[48]

* Tissues isolated from patients are shown in parentheses. Table 1 footnotes: PTA = parathyroid adenoma; PHPT = primary hyperparathyroidism; PTC = parathyroid carcinoma; PHPT = primary hyperparathyroidism; NPT = normal parathyroid; MDG = multiple gland disease.

**Table 2:** Overview of differentially expressed miRNA in gastro-entero-pancreatic neuroendocrine tumors (GEP-NETs).

Tissues/Cells	miRNAs	Potential Biological Role in GEP-NET	Platform for miRNA Expression Analysis	Internal Control for qPCR	AUC Value_ROC	Study
GEP-NET (79)	Specific site miRNA profiles (see article)	/	qPCR	/	/	[49]
GEP-NET subtypes (64)	/	/	Small RNA sequencing; qPCR	U6	/	[50]
Primary pNET (63), metastatic pNET (18), non-metastatic pNET (41)	miR-21 (↑), miR-10a (↑), miR-106b (↑)	Correlation with PanNET tumorigenesis	Bioinformatic analysis; qPCR	RNU6B	/	[51]
pNETs (6), HC (5)	28 miRNAs (see article)	/	Bioinformatic analysis from GEO data	/	/	[52]
pNET (37)	miR-3653 (↑), miR-4417 (↓), miR-574-3p (↓), miR-664b-3p (↓)	/	Microarray	/	/	[53]
pNET (57)	miR-145-5p, miR-34a-5p, miR-132-3p, miR-183-5p, miR-449a	Association with cell proliferation	qPCR	/	/	[54]
gNETs, NGM	miR-202-3p (↑)	Role in the process of dysplasia and tumor formation	Microarray; qPCR	5S rRNA	/	[55]
Hypergastrinemic patients with autoimmune atrophic gastritis and type 1 gNETs (8), HC (10), AGS_GR_ cells	miR-222 (↑)	Increased migration and actin remodeling	miRNA PCR arrays; qPCR	RNU62	/	[56]
Resected pNET (37), non-neoplastic pancreas (37), primary pNET (2), pNET with liver metastasis (2)	miR-196a (↑), miR-142-5p (↑)	Association with aggressive behavior of Pan-NETs	qPCR	U6	0.833 (miR-196a) 0.747 (miR-27b)	[57]
GEP-NET before and after SSA therapy (8)	let-7c-5p (↑), mir-24-3p (↑), mir-215-5p (↑), mir- 10a-3p (↓), mir-185-3p (↓), mir-339-5p (↓), mir-371a-5p (↓), mir-4436b- 5p (↓), mir-4653-3p (↓), mir-4793-3p (↓), mir-619-5p (↓), mir-1226-3p (↓), mir-3137 (↓), mir-4455 (↓), mir-4656 (↓)	/	Microarray; qPCR	RNU6-2_11	/	[58]
MIN6 cells	miR-17 (↑)	Role in glucose-induced β cell proliferation	qPCR	U6	/	[59]

Tissues isolated from patients are shown in parentheses. Table 2 footnotes: pNET = pancreas neuroendocrine tumor; HC = healthy controls; gNET = gastric neuroendocrine tumor; NGM= non-tumor gastric mucosa; GEP-NET = gastro-entero-pancreatic neuroendocrine tumor.

**Table 3:** Overview of differentially expressed miRNA in pituitary tumors.

Tissues/Cells	miRNAs	Potential Biological Role in Pituitary Tumors	Platform for miRNA Expression Analysis	Internal Control for qPCR	AUC Value_ROC	Study
G-PA (21), NP (3), PRL-PA (12), GH-PA (12)	miR-374b (↑), miR-17 (↑), miR-432 (↓), miR-410 (↓)	Correlation with development	miRNACHIP microarray; qPCR	RNA (U44)	/	[60]
GH-PA (13), PRL-PA (17), NF-PA (42), NP (6)	miR-34c-3p (↓), miR-34b-5p (↓), miR-338-5p (↓), miR-375 (↓), miR-493-5p (↓), miR-124-3p (↓), miR-181b-5p (↑), miR-184 (↑)	Modulation of tumor progression	NGS; qPCR	U6	/	[61]
G-PA (34), GH-PA (30), ACTH-PA (5), plurihormonal-secreting PA (3), NF-PA (3)	miR-410-3p (↑), miR-410-3p (↓)	Regulation of cell proliferation and invasiveness	qPCR	mean Ct values for miR-24 and miR-484; mean Ct values of the target gene and GAPDH	/	[62]
NF-PA (20), NP (8)	miR-524-5p (↑)	Regulation of cell proliferation, migration, invasion, and tumorigenicity	qPCR	/	/	[63]
IPA (8), non-IPA (8)	miR-132 (↑) miR-15a/16 (↑)	Suppression of cell proliferation, migration, and invasion	qPCR	U6	/	[64]
Non-invasive NF-PA (10), invasive NFPA (10)	miR-181a-5p (↑), miR-191-3p (↑)	Correlation with development	miRNA microarray; qPCR	U6	/	[65]
IPA (10), non-IPA (10)	miR-148b-3p (↓), miR-152 (↓)	Regulation of cell proliferation and invasion	qPCR	U6	/	[66]
IPA (66), non-IPA (71), HP75 cells	miR-543 (↑)	Promotion of cell proliferation, migration, and invasion	qPCR	U6	/	[67]
HP75 cells	miR-133 (↑)	Inhibition of cell migration and invasion	qPCR	/	/	[68]
PA (70), NP.	miR-26a (↑)	Onset and invasiveness	qPCR	U6	0.889 (miR-26a)	[69]
IPA (30), non-IPA (30)	miR-24 (↑), miR-34a (↑), miR-93 (↑)	Involvement in cell invasion	qPCR	U6	/	[70]
Aggressive PRL-PA (4), non-aggressive PRL-PA (8)	miR-489 (↑), let-7d* (↑), miR-138-1 * (↑), miR-520b (↓), miR-875-5p (↓), miR-671-3p (↓)	Association with aggressiveness	Bioinformatic analysis from microarray data	/	/	[71]
PA (60), NP (9)	miR-17-5p (↑)	Implication in tumorigenesis	qPCR	RNU53, U54	/	[72]
IPA (29), non-IPA (26), NP (8), AtT-20 cells	miR-106b (↑)	Regulation of cell proliferation and invasion	qPCR	U6	/	[73]
PA (50), NP (10)	miR-106b (↑)	Affect migration and invasion	qPCR	U6	/	[74]
CCA (5), IPA (13), non-IPA (9)	miR-93-5p (↑) miR-106b~25 cluster (↑)	Contribution to the acquisition of an aggressive phenotype	qPCR	U6	0.7841 (miR-106b~25) 0.8167 (MCM7 LI) 0.9133 (miR-106b~25 + MCM7 LI)	[75]
HeLa cells, AtT20 cells, *Men1^+/−^* mice, WT mice	miR-15a (↓), miR-16-1 (↓), let-7a (↓)	/	qPCR	RNU6B, SNORD95	/	[76]

* Tissues isolated from patients are shown in parentheses. Table 3 footnotes: PA = pituitary adenoma; NP = normal pituitary; G-PA = gonadotropin-secreting pituitary adenoma PRL-PA = prolactin-secreting pituitary adenoma; GH-PA = growth hormone-secreting pituitary adenoma; IPA = invasive pituitary adenoma; NF-PA = non-functioning pituitary adenoma; CCA = Crooke’s cell adenoma.

**Table 4:** Overview of c-miRNAs as candidate biomarkers in sporadic non-MEN1 associated tumors.

Biological Fluid	Study Population	c-miRNAs	Platform for miRNA Expression Analysis	Reference Genes for qPCR	AUC Value ROC	Study
Serum	Untreated siNET (21), SSA-treated siNET (21), HC (9)	miR-96 (↑), miR-182 (↑), miR-183 (↑) mIR-196a (↑), miR-200a (↑), miR-31 (↓), miR-129-5p (↓), miR-133a (↓), miR-215 (↓), miR-96 (↓), miR-182 (↓), miR-183 (↓), mIR-196a (↓), miR-200a (↓)	qPCR	miR-16	/	[80]
Plasma	Localized pancreatic cancer (15), HC (15)	miR-196a (↑), miR-1246 (↑)	qPCR	cel-miR-54	/	[81]
Serum	siNET (32), HC (25)	miR-7-5p (↑)	qPCR	cel-miR-54	0.839 (miR-7-5p)	[82]
Serum	Pituitary tumor (36), HC (8)	miR-16 (↓)	qPCR	/	/	[83]
Plasma	Different types of PA (45), HC (2)	miR-143-3p (↓)	NGS, qPCR	/	0.79 (miR-143-3p)	[84]
Serum	GH-PA (6), HC (6)	miR-423-5p (↓), miR-320a (↓)	miRScanTM Panel Chip qPCR	QB-spikein-2	/	[85]

Enrolled population is shown in parentheses. Table 4 footnotes: siNET = small intestine neuroendocrine tumor; SSA = somatostatin analogue; HC = healthy control; PA = pituitary adenoma; GH-PA = growth hormone-secreting pituitary adenoma.
